# Weaning Immunosuppressant in Patients with Failing Kidney Grafts and The Outcomes: A Single-Center Retrospective Cohort Study

**DOI:** 10.1038/s41598-020-63266-3

**Published:** 2020-04-14

**Authors:** Hyunjin Ryu, Yong Chul Kim, Jong Joo Moon, Eun Young Song, Sang-il Min, Jongwon Ha, Kwon Wook Joo, Yon Su Kim, Curie Ahn, Hajeong Lee

**Affiliations:** 1 Department of Internal Medicine, Seoul National University Hospital, Seoul, Korea; 2 Transplantation Center, Seoul National University Hospital, Seoul, Korea; 3 Department of Laboratory Medicine, Seoul National University Hospital, Seoul, Korea; 4 Departments of Surgery, Seoul National University Hospital, Seoul, Korea; 5 Transplantation Research Institute, Seoul National University College of Medicine, Seoul, Korea; 6 Kidney Research Institute, Seoul National University College of Medicine, Seoul, Korea

**Keywords:** Nephrology, Kidney diseases

## Abstract

An immunosuppressant weaning protocol in failing allografts has not yet been established. Maintaining immunosuppressants would preserve residual renal function (RRF) and prevent graft intolerance syndrome and sensitization but would increase the risks of infection and malignancy. In this study, graft failure cases after kidney transplantation in a single center were reviewed retrospectively. The outcome differences in all-cause mortality, infection-related hospitalization, cancer, graft intolerance syndrome, re-transplantation, and RRF duration between the immunosuppressant maintaining and weaning groups 6 months after graft failure were compared. Among the weaning group, the outcome differences according to low-dose steroid use were also compared at 6 and 12 months. In a total of 131 graft failure cases, 18 mortalities, 42 infection-related hospitalizations, 22 cancer cases, 11 graft intolerance syndrome cases, and 28 re-transplantations occurred during the 94-month follow-up. Immunosuppressant maintenance significantly decreased the patient survival rate 6 months after graft failure compared with weaning (log-rank *P* = 0.008) and was an independent risk factor for mortality, even after adjustments (hazard ratio, 3.01; *P* = 0.025). Infection-related hospitalization, graft intolerance syndrome development, and re-transplantation were not affected by the immunosuppressant weaning protocol. Among the immunosuppressant weaning group, low-dose steroid maintenance at 6 and 12 months helped preserved RRF (*P* = 0.008 and *P* = 0.003, respectively).

## Introduction

Kidney transplantation (KT) is currently one of the most important treatments performed to prolong the survival and improve the quality of life of patients with end-stage renal disease (ESRD)^1^. With the in-depth understanding of immunology and the development of effective immunosuppressants, the total number of KTs performed has increased rapidly. More than 84,000 KTs were performed worldwide in 2015, and there were 20,119 prevalent KT in Korea in 2018^2,3^. However, the number of patients with allograft function loss also increased rapidly owing to the increased number of accumulated KT cases and improved recipients’ survival. In the United States, approximately 5,000 KT recipients re-started dialysis owing to graft failure every year, which accounted for approximately 4–5% of incident dialysis patients^4^. In addition, 12.5–16.5% of KT recipients had re-transplantation in the United States^5^. Therefore, meticulous medical care for patients with lost allograft function is important^6^.

Patients with failed grafts have shown poor outcomes. Not surprisingly, recipients with failed grafts showed lower short-term and long-term survival rates than did those with functioning grafts^7–10^. Previous studies that used data in the United States and Canada have reported that the annual adjusted mortality rate was over three-fold higher in patients with a failed graft than in those with a functioning graft^7,11^. Additionally, they showed even higher mortality rates than did transplantation-naïve incident dialysis patients after adjusting various confounding factors, which implies that graft failure and associated factors are important for the survival of allograft recipients, even after return to dialysis^11–14^. One of the important issues associated with graft failure is when and how to wean or maintain immunosuppressants. Continuing immunosuppressants after graft failure has several advantages, such as reduction of graft intolerance syndrome occurrence^15,16^, maintenance of residual renal function (RRF)^17^, and prevention of sensitization from allografts, which could affect the outcomes of the next KT^18,19^. On the contrary, immunosuppressant maintenance could elevate the risk of infection and cardiovascular events^16,20^. Additionally, long-term use of immunosuppressants may be associated with the development of cancer^21,22^ and secondary adrenal insufficiency^23^. Therefore, a balance between the advantages and disadvantages of the use of immunosuppressants is critical in patients with failing grafts to improve their survival and the outcomes of the next KT.

However, only a few studies have been conducted in patients with failing grafts, the majority of which are retrospective cohort studies with a small number of participants and performed in non-Asian populations^16,20,24^. In this study, we aimed to explore the evidence on when and how immunosuppressants should be weaned in recipients with failing grafts.

## Materials and Methods

### Study subjects

**Figure 1:**
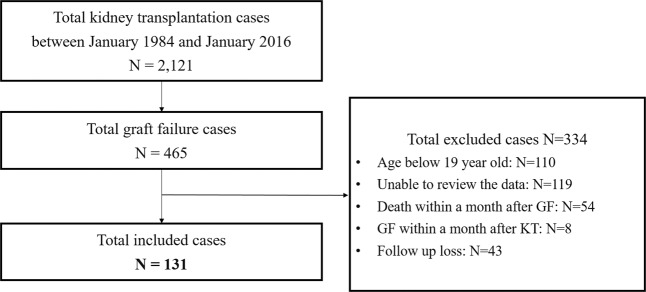
Flowsheet of the study population. After the exclusion of these 334 patients, a total of 131 patients were analyzed in this study. *Abbreviations*. GF, graft failure; KT, kidney transplantation.

This retrospective study was conducted in a single tertiary hospital. Among a total of 2,121 KTs performed in Seoul National University Hospital from January 1984 to January 2016, 465 patients lost their allograft function permanently. Allograft failure was defined as the requirement of maintenance renal replacement therapy owing to deteriorated allograft function. We excluded recipients who were aged under 19 years at the time of transplantation (n = 110), had allograft failure or mortality before 1999 (as their data could not be extracted from the electronic medical records) (n = 119), graft failure within a month after transplantation (n = 8), mortality within a month after graft failure (n = 54), and those who were lost to follow-up after transplantation (n = 43). After the exclusion of these 334 patients, a total of 131 patients were analyzed in this study (Fig. 1). This study was approved by the Institutional Review Board (IRB) of Seoul National University Hospital (IRB No. 1805-108-947) and performed in accordance with the recent guideline of the Declaration of Helsinki. Written consent was waived by the IRB because of the retrospective nature of the study with minimal risk to the study subjects.

### Clinical parameters

Electronic medical records were reviewed retrospectively. Data on the underlying disease, including diabetes and hypertension, cause of ESRD, donor type, and history of transplantation were gathered. In addition, we reviewed the duration of graft functioning and cause of allograft failure. Information on immunosuppressive treatment, including the use of steroids, before and after allograft failure was obtained.

### Definitions

We divided our subjects into two groups according to their immunosuppressant use 6 months after allograft failure as follows: immunosuppressant maintaining and immunosuppressant weaning groups. The immunosuppressant maintaining group consisted of patients receiving single steroid therapy with an equivalent dose of ≥10 mg per day of prednisolone and those using more than two kinds of concurrent immunosuppressants, including low-dose steroid with calcineurin inhibitors (CNIs) or antimetabolites, 6 months after graft failure. The immunosuppressant weaning group consisted of patients whose immunosuppressive treatment was discontinued and those receiving single steroid therapy with an equivalent dose of <10 mg per day of prednisolone (low-dose steroid therapy) 6 months after graft failure. To assess the beneficial effect of low-dose steroid use, we further subdivided the immunosuppressant weaning group according to the maintenance of low-dose steroid therapy 6 and 12 months after allograft failure as follows: steroid stopped group and steroid maintaining group.

### Outcomes

We reviewed the following outcome events classified into three groups: 1) all-cause mortality, 2) preferred immunosuppressant withdrawal outcomes (infection-related hospitalization and cancer occurrence), and 3) preferred immunosuppressant maintenance outcomes (graft intolerance syndrome development, re-transplantation, and RRF duration, defined as the duration of diuretic therapy after graft failure).

### Statistical analysis

Statistical analyses were performed using SPSS (version 21.0; SPSS Inc.), except for the survival analysis. All continuous variables showed a non-normal distribution. Therefore, they were presented as medians (interquartile ranges [IQRs]). The chi-square test and Mann-Whitney test were used to compare the baseline characteristics and outcomes between the immunosuppressant maintaining and weaning groups. The same methods were used to compare the basic characteristics and outcomes between the steroid maintaining and weaning groups 6 and 12 months after graft failure in the subgroup analysis of the immunosuppressant weaning group. *P*-values of <0.05 were interpreted as statistically significant. Kaplan-Meier curves and the log-rank test were employed using R version 3.4.0 (Foundation for Statistical Computing, Vienna, Austria) to determine the difference in the incidence of outcomes between the immunosuppressant weaning and maintaining groups. To analyze the independent effect of immunosuppressant maintenance 6 months after graft failure on mortality, a Cox regression analysis was conducted by adjusting for sex, age at graft failure, type of KT, presence of diabetes or hypertension, re-transplantation, and dialysis modality after graft failure.

## Results

### Baseline characteristics

A total of 131 patients with allograft failure were analyzed. Thirty-four (26%) patients were women, and the median age at the time of KT was 33 [IQR, 19–70] years. Eighteen (13.7%) and 130 (99.2%) patients had diabetes and hypertension, respectively. The major proven causes of ESRD were chronic glomerulonephritis (n = 51, 38.9%), diabetes (n = 16, 12.2%), and vesicoureteral reflux (n = 9, 6.9%); the cause was unknown in 53 (40.5%) patients. Seventeen (13%) patients received their kidney from a deceased donor, and six (4.6%) received a second allograft.

The median graft survival duration was 127 [IQR, 70–162] months, and the mean age at the time of allograft failure was 44.9 ± 11.1 years. The most common cause of allograft failure was rejection (n = 82, 62.6%), followed by recurred or *de novo* glomerulonephritis (n = 29, 22.1%), which were diagnosed with renal biopsy. Other systemic causes of allograft failure were allograft kidney cancer (n = 3), acute tubular injury (n = 2), septic shock (n = 1), cytomegalovirus infection (n = 1), ischemic nephropathy (n = 1), renal artery aneurysm (n = 1), and acute decompensated heart failure (n = 1). In five (3.8%) patients, we could not find a definite cause of allograft failure. Twenty-nine (22.1%) patients received high-dose steroid pulse therapy within 12 months before graft failure (median, 107 [IQR, 36–156] days) to treat the following causes: acute rejection (n = 16), recurrent glomerulonephritis (n = 5), chronic antibody-mediated rejection (n = 4), chronic allograft nephropathy (n = 2), and unknown (n = 2). Among them, 18 patients received additional immunosuppressive therapy, such as administration of OKT3 (n = 2), anti-thymocyte globulin (n = 7), intravenous immunoglobulin G (n = 7), rituximab (n = 7), bortezomib (n = 1), and plasmapheresis (n = 6). After graft failure, peritoneal dialysis was started in 22 (16.8%) patients as their maintenance renal replacement therapy; hemodialysis in 106 (80.9%) patients; and combination of peritoneal dialysis and hemodialysis in 3 patients (Table 1).

**Table 1:** Baseline characteristics of the study subjects.

	Total
Number of cases	131
Age at kidney transplantation (years)^a^	33 [19;70]
Female (%)	34 (26)
Diabetes (%)	18 (13.7)
Hypertension (%)	130 (99.2)
Cause of end stage renal disease (%)	
Diabetes	16 (12.2)
Hypertension	2 (1.5)
Chronic glomerulonephritis	51 (38.9)
Other	9 (6.9)
Unknown	53 (40.5)
Deceased donor kidney transplantation (%)	17 (13)
2^nd^ kidney transplantation (%)	6 (4.6)
Graft survival (months)^a^	127 [70;162]
Age at graft failure (year)^b^	44.9 ± 11.1
Cause of graft failure (%)	
Rejection	70 (53.4)
Non-compliance	12 (9.2)
Recurred glomerulonephritis	29 (22.1)
Others	20 (15.3)
History of immunosuppressant pulse therapy before graft failure within 1 year (%)	29 (22.1)
Peritoneal dialysis as post graft failure dialysis modality (%)	25 (19.1)
Patients survival duration after kidney transplantation (months)^a^	225 [162;294.5]
Outcome duration after kidney transplantation (months)^a^	174 [129.5;239]

^a^Represented as median and [interquartile ranges] and ^b^represented as mean ± standard deviations.

### Outcomes after graft failure

During a median follow-up duration of 94 [IQR, 58–144.5] months after graft failure, 18 (13.7%) patients eventually died due to 8 cardiovascular events, 4 infections, 3 malignancies, 1 acute renal failure due to rhabdomyolysis, 1 colon perforation and 1 unknown reason, respectively.

A total of 71 infection-related hospitalizations occurred in 42 (32.1%) patients regarding the preferred immunosuppressant withdrawal outcomes. The median time to hospitalization from graft failure was 22 [IQR, 6.5–57] months. The most common cause of infection-related admission were pneumonia in 15 (11.4%) patients, and soft tissue infections in 15 (11.4%), followed by catheter-related or permanent vascular access-related infections in 12 (9.2%), peritoneal dialysis-related peritonitis in 10 (7.6%), gastrointestinal infections in 9 (6.8%), viral infections in 5 (3.8%), urinary tract infections in 3 (2.3%), and unknown-origin infections in 2 (1.5%). Twenty-two (16.8%) patients developed new-onset cancer after allograft failure invading a variety of organs, such as the genitourinary tract (n = 7), gastrointestinal tract (n = 6), thyroid (n = 3), lymphoma (n = 2), skin (n = 1), breast (n = 1), cervix (n = 1), and Kaposi’s sarcoma (n = 1).

In terms of the preferred immunosuppressant maintaining outcomes, graft intolerance syndrome occurred in 11 (8.4%) patients, and 9 (6.8%) patients eventually needed graft nephrectomy; however, 2 (1.5%) cases subsided without nephrectomy. A total of 28 (21.4%) patients underwent re-transplantation after graft failure. The RRF was maintained for a median of 6 [IQR, 1–16] months based on the duration of diuretic therapy.

### Weaning immunosuppressants and its impact on clinical outcomes

The weaning protocol varied among the patients. CNIs were weaned before antimetabolites in 42 (32.1%) patients, antimetabolites before CNIs in 62 (47.3%), and both CNIs and antimetabolites simultaneously in 24 (18.3%). In most cases, the steroid was weaned last, except in 1 patient wherein CNIs were weaned last.

**Figure 2:**
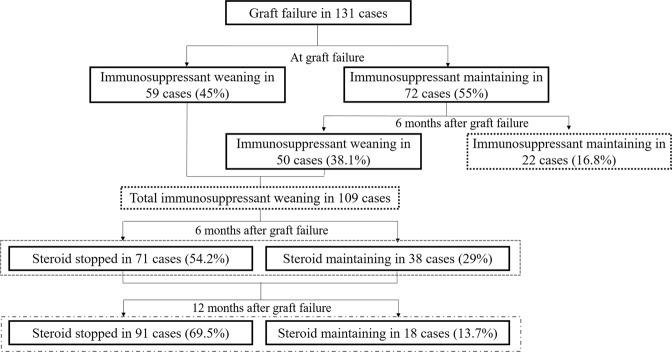
Immunosuppressant weaning protocols in the study population. At 6 months after graft failure, immunosuppressant maintained in 22 cases (16.8%) and weaned in 109 cases (83.2%). Among the immunosuppressant weaned cases, low-dose steroid was maintained in 38 cases (29%) and 18 cases (13.8%) at 6 month and 12 month after graft failure, respectively. *Abbreviations*. ISA, immunosuppressant.

At the time of graft failure, immunosuppressants were maintained in 72 (55%) patients: triple therapy with CNIs, antimetabolites, and steroids in 25 (34.7%); CNIs and steroids in 30 (41.7%); antimetabolites and steroids in 13 (18.1%); CNIs only in 1 (1.4%); and steroids only in 3 (4.2%). Immunosuppressants were weaned at the time of allograft failure in 59 (45%) patients; 49 (83.1%) used steroids only, and 10 (16.9%) stopped taking all immunosuppressants before graft failure. Six months after allograft failure, immunosuppressants were maintained in 22 (16.8%) patients: triple therapy in 8 (36.4%), CNIs and steroids in 11 (50%), and antimetabolites and steroids in 2 (9.1%) and steroid only in 1 (4.5%). Conversely, immunosuppressants were weaned 6 months after graft failure in 109 (83.2%) patients: 38 (34.9%) received low-dose steroid therapy only, and 71 (65.1%) stopped taking all immunosuppressants. Among the immunosuppressant weaning group 6 months after graft failure, a total of 91 (69.5%) patients stopped taking all immunosuppressants; however, 18 (13.7%) still received low-dose steroid therapy until 12 months after graft failure (Fig. 2).

In the comparison between the immunosuppressant maintaining and weaning groups 6 months after graft failure, there was no significant difference in the baseline characteristics, clinical outcomes, renal replacement therapy modality after graft failure, and duration of patient survival, diuretic use, and follow-up after graft failure. However, several mortality events occurred in the immunosuppressant maintaining group (n = 7, 27.3%: infection [n = 2], cardiovascular events [n = 2], cancer [n = 2], and rhabdomyolysis [n = 1]) compared with those in the weaning group (n = 11, 10.1%: cardiovascular events [n = 6], infection [n = 2], cancer [n = 1], colon perforation [n = 1], and unknown cause [n = 1]; *P* = 0.014; Table 2).

**Table 2:** Basic characteristics and outcomes difference according to immunosuppressant usage at 6 months after graft failure.

	Immunosuppressant weaning 6 months after graft failure	Immunosuppressant maintaining 6 months after graft failure	*P*-values
Number of cases	109	22	0.861
Age at kidney transplantation (year old)	33 [28;42]	33.5 [29;39]	0.863
Female (%)	30 (27.5)	4 (18.2)	0.362
Diabetes (%)	16 (12.2)	2 (1.5)	0.736
Hypertension (%)	108 (99.1)	22 (100)	1
Cause of end stage renal disease (%)			0.316
Diabetes	14 (12.8)	2 (9.1)	
Hypertension	2 (1.8)	0	
Chronic glomerulonephritis	39 (35.8)	12 (54.5)	
Other	7 (6.4)	2 (9.1)	
Unknown	47 (43.1)	6 (27.3)	
Deceased donor kidney transplantation (%)	16 (17.4)	1 (5.9)	0.303
2^nd^ kidney transplantation (%)	6 (6.5)	0	0.589
Graft survival (months)^a^	120 [68;159]	143 [114;1]	0.136
Age at graft failure (year old)	44.6 ± 11.5	46.4 ± 9.0	0.479
Cause of graft failure (%)			0.768
Rejection	59 (54.1)	11 (50.0)	
Non-compliance	11 (10.1)	1 (4.5)	
Recurred glomerulonephritis	21 (19.3)	8 (36.4)	
Others	18 (16.5)	3 (7.7)	
History of immunosuppressant pulse therapy before graft failure within 1 year(%)	21 (22.8)	8 (20.5)	0.771
Peritoneal dialysis as post graft failure dialysis modality (%)	19 (17.4)	6 (27.3)	0.439
All-cause mortality (%)	11 (10.1)	7 (31.8)	0.014
Immunosuppressant withdrawal preferred outcomes			
Hospitalization due to infection (%)	36 (33)	6 (27.3)	0.782
Cancer (%)	17 (15.6)	5 (22.7)	0.531
Immunosuppressant maintenance preferred outcomes			
Graft intolerance syndrome (%)	10 (9.2)	1 (4.5)	0.69
Nephrectomy due to graft intolerance syndrome (%)	9 (8.3)	0	0.355
Re-transplantation (%)	25 (22.9)	3 (13.6)	0.406
Diuretics usage duration after graft failure (months)^a^	3 [0;14]	8.5 [6;17]	0.113
Outcome duration (months)^a^	45 [18;86]	29.5 [15;68]	0.508
Patients survival duration after kidney transplantation (months)^a^	231 [165;292]	224.5 [154;334]	0.635
Follow up duration after graft failure (months)^a^	97 [65;144]	78.5 [40;151]	0.385

^a^Represented as median and [interquartile ranges]. *P*-value from the chi-square test and Mann-Whitney test.

**Figure 3:**
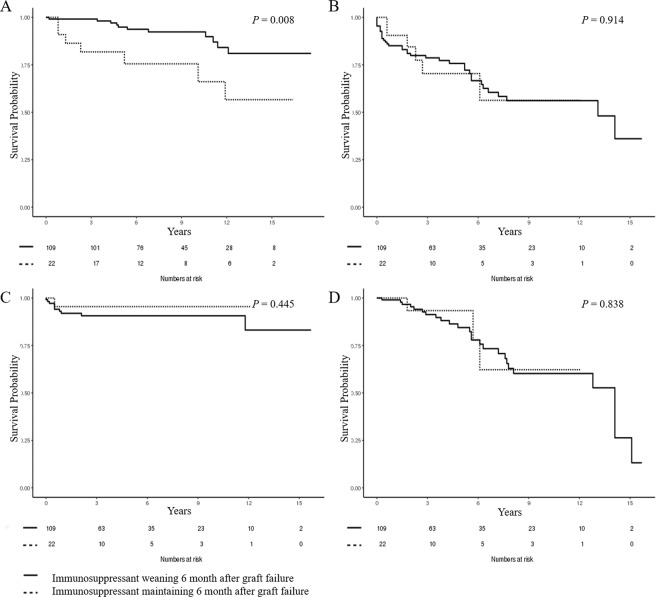
Kaplan-Meier curve of the outcomes in immunosuppressant weaning and maintaining groups 6 months after graft failure. (**A**) all-cause mortality, (**B**) hospitalization due to infection, (**C**) graft intolerance syndrome and (**D**) re-transplantation. Immunosuppressant maintaining group showed significantly lower survival rates than weaning group (*P* = 0.008). However, there was no statistically significant difference in infection-related hospitalization (*P* = 0.914), graft intolerance syndrome (*P* = 0.445), and re-transplantation (*P* = 0.838).

The survival analysis using the Kaplan-Meier curves and log-rank test also showed significantly lower survival rates in the immunosuppressant maintaining group than in the immunosuppressant weaning group (log-rank *P* = 0.008). Moreover, the elevated mortality risk remained even after adjustment for sex, age at the time of graft failure, donor type, presence of diabetes or hypertension, re-transplantation, and dialysis modality after graft failure (adjusted hazard ratio, 3.01; 95% confidence interval, 1.15–7.88; *P* = *0.025*). However, there were no significant differences in the other preferred immunosuppressant withdrawal outcomes, such as infection-related hospitalization (log-rank *P* = 0.914), nor in the preferred immunosuppressant maintaining outcomes, such as graft intolerance syndrome occurrence (log-rank *P* = 0.445) and re-transplantation (log-rank *P* = 0.838), between the two groups (Fig. 3).

### Effects of low-dose steroid maintenance 6 and 12 months after graft failure

The subgroup analysis among the immunosuppressant weaning group was conducted according to the duration of steroid therapy. Among the 109 patients in whom immunosuppressants were weaned 6 months after graft failure, 71 (54.2%) stopped taking steroids, while 38 (34.9%) continued taking them in low dose. Twelve months after graft failure, 91 (69.5%) patients stopped taking steroids, while 18 (16.5%) still received low-dose steroid therapy (Fig. 2). There was no significant difference in the outcomes after graft failure, including all-cause mortality, infection-related hospitalization, post-transplantation cancer occurrence, graft intolerance syndrome occurrence, and re-transplantation between the steroid weaning and maintaining groups both 6 and 12 months after graft failure. The steroid weaning group had a higher incidence of nephrectomy due to graft intolerance syndrome both 6 and 12 months after graft failure than the steroid maintaining group, although the difference was not significant (*P* = 0.158 and *P* = 0.351, respectively). The duration of diuretic therapy was longer in the steroid maintaining group both 6 (*P* = 0.008) and 12 months after graft failure (*P* = 0.003) (Table 3).

**Table 3:** The outcome differences according to low dose steroid usages at 6 month and 12 month after graft failure in the subgroup analysis of immunosuppressant weaning group at 6 months after graft failure.

	Steroid stopped 6 months after graft failure	Steroid maintaining 6 months after graft failure	*P*-values	Steroid stopped 12 months after graft failure	Steroid maintaining group 12 months after graft failure	*P*-values
**Number of cases**	71	38		91	18	
**All-cause mortality (%)**	10 (14.1)	1 (2.6)	0.093	10 (11)	1 (5.6)	0.687
**Immunosuppressant withdrawal preferred outcomes**						
Hospitalization due to infection (%)	24 (33.8)	12 (31.6)	0.983	29 (31.9)	7 (38.9)	0.761
Cancer (%)	12 (16.9)	5 (13.2)	0.813	14 (15.4)	3 (16.7)	1.0
**Immunosuppressant maintaining preferred outcomes**						
Graft intolerance syndrome (%)	8 (11.3)	2 (5.2)	0.489	9 (9.9)	1 (5.6)	1.0
Nephrectomy due to graft intolerance syndrome (%)	8 (11.3)	1 (2.6)	0.158	9 (9.9)	0	0.351
Re-transplantation (%)	18 (25.4)	7 (18.4)	0.561	20 (22)	5 (27.8)	0.555
Diuretics usage duration after graft failure (months)^a^	1 [0;11]	9 [2;32]	0.008	2 [0;9.5]	25 [6;38]	0.003

^a^Represented as median and [interquartile ranges]. *P*-value from the chi-square test and Mann-Whitney test.

## Discussion

In this study, we discovered that maintaining immunosuppressants 6 months after graft failure elevated the risk of all-cause mortality approximately three-fold compared with weaning immunosuppressants, even after adjusting for other confounding factors, although the other outcomes were not significantly affected. Conversely, we suggest that maintaining low-dose steroids until 12 months after graft failure could preserve RRF, which was based on the duration of diuretic therapy. Based on these findings, CNIs and antimetabolites may be weaned within 6 months after graft failure, and low-dose steroids may be maintained up to 12 months after graft failure for survival improvement and RRF preservation in patients who have lost their allograft function.

Maintaining immunosuppressants after graft failure has both advantages and disadvantages. It influences the outcomes of patients with failing grafts in both positive and negative aspects. An appropriate immunosuppressant weaning protocol is important to balance its positive and negative effects and consequently improve the overall outcomes of patients with allograft failure. There are only a few recommendations and guidelines regarding immunosuppressive therapies in patients with failing grafts^3,23,25^. In addition, there has been no definite immunosuppressant weaning protocol in KT recipients until recently. The consensus from currently available guidelines and recommendations suggest weaning immunosuppressants 6 months after graft failure, especially in patients with minimal RRF. It is recommended to taper steroids carefully and gradually while monitoring patients’ symptoms for adrenal insufficiency and rejection^25^. Our study findings also support the recommendation of early withdrawal of immunosuppressants within 6 months after graft failure. However, our data suggest that maintaining steroids in low doses (equivalent dose of <10 mg per day of prednisolone) up to 12 months can be beneficial in preserving RRF.

In our study, immunosuppressant maintenance 6 months after graft failure increased the risk of all-cause mortality even after adjusting for other confounding factors. In a previous study that used USRDS data, the main cause of mortality in patients with graft failure was cardiovascular problems and infections^8^. In other studies, increased risks of infection and hospitalization were associated with immunosuppressant maintenance after graft failure^16,20^. The study also showed that the main cause of mortality was cardiovascular complications (44.4%), followed by infections (22.2%). However, the other outcomes related to the adverse effects of immunosuppressants, including infection-related hospitalization and post-KT cancer occurrence, did not significantly differ between the immunosuppressant maintaining and weaning groups. This discrepancy between our results and those of previous studies might be attributed to the small number of both infection-related hospitalization (32.1%) and study subjects, especially in the immunosuppressant maintaining group 6 months after graft failure; there were only 22 (16.8%) patients included. Further, among the 42 patients who had infection-related hospitalization in this study, 16 (38.1%) had more than two hospitalizations. As the survival analysis was conducted using the first hospitalization data, the increased risk of infection in immunosuppressant maintenance might have been devaluated. The rates of graft intolerance syndrome occurrence and nephrectomy, which are considerable side effects of immunosuppressant weaning, did not differ between the immunosuppressant weaning and maintaining groups in this study.

One of the strengths of this study is that a subgroup analysis was conducted among the immunosuppressant weaning group according to low-dose steroid maintenance 6 and 12 months after graft failure. In the subgroup analysis, the steroid maintaining group 6 and 12 months after graft failure showed a significantly longer use of diuretics, which was also interpreted as the duration of RRF. It is well known that RRF is important in improving the survival and quality of life of incidence dialysis patients with naïve kidneys^8,9,13,26^. The importance of RRF on survival has also been reported in peritoneal dialysis patients with graft failure^17^. However, in this study, no survival benefit was observed in the steroid maintaining group, although RRF was preserved longer in this group than in the immunosuppressant weaning group. Additionally, the rate of nephrectomy due to graft intolerance syndrome was lower in the steroid maintaining group 6 and 12 months after graft failure than in the immunosuppressant weaning group although the difference was not significant (*P* = 0.158 and *P* = 0.351, respectively). Furthermore, maintaining low-dose steroids did not increase the adverse outcomes of mortality, infection-related hospitalization, and post-KT cancer occurrence. Therefore, maintaining low-dose steroids until 12 months after graft failure may have beneficial effects on RRF without increasing adverse events.

None of the patients had nephrectomy due to graft intolerance syndrome in the steroid maintaining group 12 months after graft failure, although this number did not significantly differ with that in the steroid stopped group (n = 9 patients, 9.9%). To determine the risk factor for graft intolerance syndrome in our study patients, we compared the immunosuppressant weaning protocol between the patients with (n = 11, 8.4%) and without graft intolerance syndrome (n = 120, 91.6%). There was no significant difference in the immunosuppressant and steroid weaning protocols between the two groups (Table S1). As the number of graft intolerance syndrome cases in our study was relatively too small, we could not conclude whether immunosuppressant weaning could increase the risk for graft intolerance syndrome after graft failure.

This study has a few limitations. Among the 465 patients with graft failure, we could only investigate 131 (28.2%) patients in this study. Further, the number of analyzed patients and events was small, which might have lessened the statistical power. The dose of CNI or antimetabolites after graft failure was not included in the analysis which could be a confounding factor. The direct residual urine output could not be assessed; instead, RRF was considered based on the duration of diuretic therapy after graft failure since diuretics were only prescribed when the patients had residual urine output in the studied center. We could also not assess the effects of immunosuppressant weaning on allosensitization, owing to the retrospective nature of the study. Only 19% of the patients with graft failure had sensitization data both before and after the graft failure. However, this study also has strengths, one of which is that it was conducted in a single-center where the study population received relatively homogeneous medical management. Further, the various outcomes of the patients with graft failure (i.e., preferred immunosuppressant withdrawal and maintaining outcomes) were reviewed in detail. By conducting a subgroup analysis according to steroid use, the possible beneficial effect of low-dose steroid maintenance could be reported. Nevertheless, larger prospective cohort studies are needed to establish evidence for optimizing and personalizing immunosuppressant weaning protocols to improve the outcomes of patients with graft failure.

## Conclusion

Considering the increasing number of patients with allografts and their improving survival, balancing the favorable and adverse effects of immunosuppressants is crucial in patients with graft failure to improve their outcomes and prepare them for their next KT. Along with current recommendations, our data suggest that immunosuppressants should be tapered within 6 months after graft failure. However, patients with RRF can benefit from low-dose steroid maintenance until 12 months after graft failure without increasing adverse outcomes. Further larger prospective cohort studies are required to establish evidence for developing immunosuppressant weaning protocols in graft failure patients.

## Supplementary information

Supplementary information.

## Data Availability

All produced data are available as upon request.
