# Supplementary material for: Weaning Immunosuppressant in Patients with Failing Kidney Grafts and The Outcomes: A Single-Center Retrospective Cohort Study
Source: Sci Rep. 2020 Apr 14;10:6425. doi: 10.1038/s41598-020-63266-3 (PMC7156393; doi:10.1038/s41598-020-63266-3)
Supplement: Supplementary file 1 — Supplementary information. [file 41598_2020_63266_MOESM1_ESM.docx]

**Table S1. Difference in immunosuppressant weaning protocols according to graft intolerance syndrome occurrence during the follow ups after graft failure**

|  | Graft intolerance syndrome (+) | Graft intolerance syndrome (-) | *P*-values |
| --- | --- | --- | --- |
| Number of cases | 11 | 120 |  |
| Weaned immunosuppressant at graft failure, (%) | 4 (36.4) | 68 (56.7) | 0.221 |
| Weaned immunosuppressant at 1 month after graft failure, (%) | 3 (27.3) | 54 (45) | 0.347 |
| Weaned immunosuppressant at 3 month after graft failure, (%) | 2 (18.2) | 37 (30.8) | 0.505 |
| Weaned immunosuppressant at 6 month after graft failure, (%) | 1 (9.1) | 21 (17.5) | 0.690 |
| Maintained low dose steroids at 6 month after graft failure, (%) | 3 (27.3) | 57 (47.5) | 0.197 |
| Maintained low dose steroids at 12 month after graft failure, (%) | 1 (9.1) | 32 (26.7) | 0.289 |
| Low dose steroids maintained durations, (months)^a^ | 4 [0.25-9.75] | 6 [0.75-17.5] | 0.472 |

^a^ Represented as median and [ranges]. *P*-value from the chi-square test and Mann-Whitney test.
